# Nachhaltigkeit in der Intensiv- und Notfallversorgung: Eine bundesweite Umfrage von der Deutschen Gesellschaft für Internistische Intensivmedizin und Notfallmedizin

**DOI:** 10.1007/s00063-023-01039-2

**Published:** 2023-06-21

**Authors:** Jorge Garcia Borrega, Carsten Hermes, Victoria König, Valery Kitz, Sverrir Möller, Dominik Stark, Uwe Janssens, David Mager, Matthias Kochanek

**Affiliations:** 1 Klinik I für Innere Medizin, Zentrum für Integrierte Onkologie Aachen Bonn Köln Düsseldorf, Uniklinik Köln, Kerpener Str. 62, 50937 Köln, Deutschland; 2 Hochschule für angewandte Wissenschaften (HAW), Hamburg, Deutschland; 3 Akkon Hochschule für Humanwissenschaften, Berlin, Deutschland; 4 Hamburg, Deutschland; 5 Interdisziplinäre Intensivstation, Pflegeentwicklung, Agaplesion Diakonieklinikum Hamburg, Hohe Weide 17, 20259 Hamburg, Deutschland; 6 Interdisziplinäre konservative Intensivstation, Universitätsklinikum Schleswig-Holstein (UKSH), Lübeck, Ratzeburger Allee 160, 23538 Lübeck, Deutschland; 7 Innere Medizin und Internistische Intensivmedizin, St-Antonius-Hospital gGmbH, Eschweiler, Deutschland; 8 Krankenhaus der Barmherzigen Brüder Trier, Trier, Deutschland

**Keywords:** Zukunft, Intensivmedizin, Notfallmedizin, Befragung, Klimawandel, Future, Critical care, Emergency medicine, Questionnaire, Climate change

## Abstract

**Hintergrund:**

Die Auswirkungen des Klimawandels auf den Menschen sind bekannt. Das Gesundheitssystem trägt je nach Land mit zwischen 5 % und 7 % der Treibhausgasemissionen auch selbst einen relevanten Anteil dazu bei und eine Anpassung zu einem nachhaltigeren Arbeiten ist nötig.

**Ziel der Arbeit:**

Die Umfrage untersucht, ob Nachhaltigkeit im Krankenhaus und speziell im Bereich der Notfall- und Intensivversorgung eine Rolle spielt. Erfragt wurden auch konkrete Maßnahmen zur Nachhaltigkeit und welche Hürden vorhanden sind.

**Material und Methoden:**

Die AG Nachhaltigkeit der Deutschen Gesellschaft für Internistische Intensivmedizin und Notfallmedizin (DGIIN) führte eine elektronische Befragung unter dem Personal von Intensivstationen, Notaufnahmen und Rettungsdiensten in Deutschland durch.

**Ergebnisse:**

In die Auswertungen wurden 218 Umfrageergebnisse eingeschlossen. Insgesamt 108 (50 %) Teilnehmende kamen aus dem Pflegebereich und 98 (45 %) gehören dem ärztlichen Personal an. Die Mehrzahl der Teilnehmenden arbeitet auf einer Intensivstation (181 [83 %]) gefolgt von der Intermediate-Care-Station (52 [24 %]). 104 (47 %) Teilnehmende gaben an, dass ihre Arbeitsstätte schon Maßnahmen zur Nachhaltigkeit umgesetzt hat. Die Einschätzung, ob die Geschäftsführung das Thema Nachhaltigkeit in ihre Entscheidungen einfließen lässt, wurde bei nur 20 % angegeben. Potenzial für Verbesserung werden unter anderen im Energie- und Abfallmanagement gesehen.

**Diskussion:**

Die Umfrageergebnisse zeigen, dass 1. eine hohe Motivation der Mitarbeitenden besteht, sich mit dem Thema Nachhaltigkeit auseinanderzusetzen und Maßnahmen umzusetzen, und 2. das Potenzial, ein ressourcenschonendes und umweltfreundliches Krankenhaus zu etablieren, längst nicht ausgeschöpft ist. 3. Es muss Priorität werden, dass Entscheidungsträger:innen im Krankenhaus Nachhaltigkeit propagieren, Prozesse transparent gestalten und die Motivation der Mitarbeitenden zum Thema Nachhaltigkeit unterstützen. Darüber hinaus muss dieser Prozess von Politik und Gesundheitskassen mitgetragen werden.

## Abstract (en)

**Background:**

The impact of climate change on humans is well known. However, the health care system is also a relevant contributor, accounting for up to 5–7% of global greenhouse gas emissions, and work should be adapted to be more sustainable.

**Aim:**

The survey investigated whether sustainability plays a role in hospitals and specifically in the field of emergency and intensive care. Concrete measures and which hurdles are already recognized were also inquired.

**Materials and methods:**

The “AG Nachhaltigkeit” (working group on sustainability) of the “Deutschen Gesellschaft für Internistische Intensivmedizin und Notfallmedizin” (DGIIN) conducted an electronic survey among the staff of intensive care units, emergency rooms, and ambulance services in Germany.

**Results:**

In all, 218 survey results were included in the analysis: 108 (50%) participants were from the nursing sector and 98 (45%) belonged to the medical staff. The majority of participants work in an intensive care unit (181 [83%]) followed by intermediate care unit (52 [24%]). A total of 104 (47%) participants indicated that their workplace had already implemented sustainability measures. However, when asked whether decision-makers in the workplaces incorporate sustainability into their decisions, management scored highest with only 20%. Potential for improvement is seen in energy and waste management, among others.

**Conclusion:**

The survey results show that (1) employees are highly motivated to address the issue of sustainability and to implement measures, (2) the potential to establish a resource-saving and environmentally friendly hospital is far from being exhausted, and (3) it must become a priority that decision-makers in the hospital propagate sustainability, make processes transparent, and support the motivation of employees on the subject of sustainability. In addition, this process must be supported by politicians and health insurance companies.

## Einleitung

Obwohl die Auswirkungen von Umweltverschmutzung und des Klimawandels auf den Menschen bekannt und spürbar sind, wurde den Auswirkungen des Gesundheitswesens auf den Klimawandel bisher wenig Aufmerksamkeit geschenkt [6, 16].

Zum einen müssen die Auswirkungen und die damit verbundenen Anforderungen des Klimawandels bewältigt werden (Anstieg der Erkrankten durch extreme Wetterereignisse, Pandemien oder die Ausbreitung infektiöser Krankheiten) [2, 8, 9, 12]. Zum anderen ist der Gesundheitssektor für 5 –7 % der globalen Treibhausgasemissionen verantwortlich [5, 14].

Bis jetzt galt es in der Medizin und insbesondere in der Intensiv- und Notfallversorgung mittels Einsatzes von Personal und Material, Patient:innen zu behandeln und Menschenleben zu retten. Dies kann auf Dauer nicht ressourcenschonend und umweltverträglich sein. Dies zeigt sich beispielsweise an den Vereinigten Staaten von Amerika (USA): Hier liegt der Anteil der Gesundheitsausgaben am Bruttoinlandsprodukt (BIP) bei über 17 % und damit weit höher als in anderen Ländern. Diese Ausgaben für das US-amerikanische Gesundheitswesen führen zu hohen Treibhausgasemissionen (10 % der gesamten US-amerikanischen Gesundheitsausgaben; [4]).

Obwohl der Begriff der „Nachhaltigkeit“ nicht eindeutig definiert ist, versteht man im Allgemeinen darunter, den Bedürfnissen der Gegenwart so gerecht zu werden, dass zukünftige Generationen nicht eingeschränkt werden. Nachhaltigkeit soll dabei wirtschaftlich effizient, sozial gerecht und ökologisch tragfähig sein. Da die Anforderungen an Technik, Personal und Material in der Medizin einen sensiblen Bereich betreffen, stellt Nachhaltigkeit hier eine besondere Herausforderung dar.

Die vorliegende Umfrage untersuchte, ob und in welchem Ausmaß Nachhaltigkeit im Krankenhaus und speziell im Bereich der Notfall- und Intensivversorgung eine Rolle spielt. Es wurde erfragt, welche konkreten Maßnahmen schon umgesetzt sind, welche Hürden bereits erkannt werden und was nachhaltiges Arbeiten im täglichen Alltag einer Intensivstation und Notaufnahme bedeuten könnte.

## Methode

### Studiendesign und Entwicklung der Umfrage

Die Arbeitsgruppe Nachhaltigkeit der Deutschen Gesellschaft für Internistische Intensivmedizin und Notfallmedizin (DGIIN) führte eine anonyme, elektronische Querschnittsbefragung unter dem Personal von Intensivstationen, Notaufnahmen und Rettungsdiensten in Deutschland durch. Der Fragebogen wurde auf der Grundlage aktueller Literatur durch die Autor:innen selbst erstellt. Multi-Multiple-Choice- und Freitextfragen sowie modifizierte Likert-Skalen wurden verwendet. Die Umfrage umfasste 43 Fragen, die in 8 Abschnitte unterteilt waren. Die Umfrage wurde im Vorfeld mit zufällig ausgewählten Pflegefachpersonen und Ärzt:innen getestet.

### Umfrageteilnehmende und Datenerhebung

Die Umfrage richtete sich an Mitarbeitende im Rettungsdienst, Pflegefachpersonen und Ärzt:innen, die im Rettungsdienst, Notfallaufnahmen und Intensivstationen arbeiten. Die Umfrage wurde per E‑Mail, Newsletter sowie über verschiedene Kanälen der sozialen Medien und der DGIIN-Homepage verbreitet. Es wurden keine personenidentifizierbaren Daten erhoben, IP-Adressen wurden nicht erfasst und das Ausfüllen der Umfrage implizierte die Zustimmung zur Umfrage. Die Umfrage war vom 15.12.2022 bis zum 27.02.2023 online und wurde über die Umfrageplattform LimeSurvey® (LimeSurvey Community Edition, Version 3.28.47+230131; Host: Rechenzentrum der Universität zu Köln, Köln, Deutschland) erfasst. Ein Ethikantrag wurde nicht gestellt, da keine Beteiligung von Patient:innen oder die Nutzung von Patient:innendaten stattgefunden hat.

### Statistische Auswertung

Die Daten wurden auf Vollständigkeit und Duplikate geprüft und bereinigt. Die statistische Analyse wurde mit Microsoft Excel Version 2303 (Redmond, WA, USA) durchgeführt und die Diagramme wurden teilweise mit SankeyMATIC (OpenSource) erstellt.

Eine deskriptive Statistik wurde für die Darstellung der Daten verwendet. Die Daten werden als Anzahl und relative Häufigkeit oder als Median und 25–75 %-Interquartilsbereich (IQR) dargestellt. Offene Antworten wurden kategorisiert ausgewertet.

## Ergebnisse

### Basisdaten

An der Umfrage haben insgesamt 276 Personen teilgenommen. In die Auswertungen wurden anschließend nur Umfrageergebnisse eingeschlossen, die mindestens 90 % der Fragen beantwortet hatten (*n* = 218). Durch den offenen Verteiler über verschiedene Medien und Social-Media-Kanäle kann eine Rücklaufquote nicht ermittelt werden.

**Figure 1:**
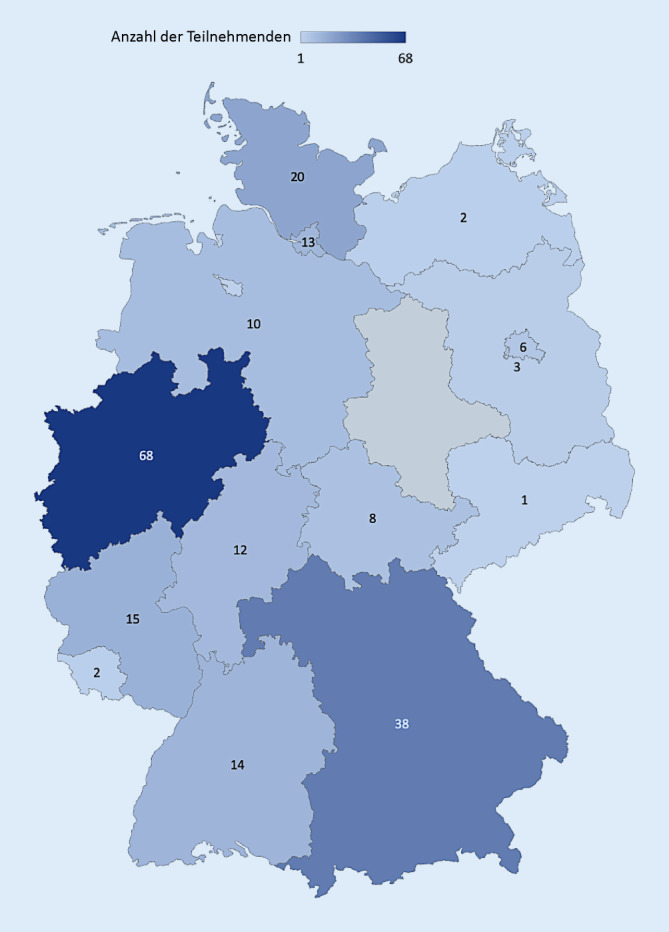

Das mediane Alter lag bei 40,5 Jahre (IQR 32–49) mit fast gleicher Beteiligung von Frauen und Männern. Im Durchschnitt hatten die Teilnehmenden eine Berufserfahrung von 15 Jahren (IQR 7–22). 108 (50 %) Teilnehmende kamen aus dem Pflegebereich und 98 (45 %) gehören dem ärztlichen Personal an. Die Mehrzahl der Teilnehmenden arbeiten auf einer Intensivstation (181 [83 %]) gefolgt von Intermediate-Care-Station (52 [24 %]; Mehrfachnennung möglich). Der Großteil (96 [45 %]) der Teilnehmenden gab an, in einem Krankenhaus mit > 1000 Betten zu arbeiten. Die meisten Teilnehmenden arbeiten in einem Krankenhaus in öffentlicher Trägerschaft (150, [68,8 %]). Die Verteilung nach Bundesländern ist in Abb. 1 dargestellt. Weitere Charakteristika kann man der Tab. 1 entnehmen.

**Table 1:** 

Charakteristika der Teilnehmenden	*n*	%
Beteiligte insgesamt ( 85 % aller Fragen)	218	–
Alter in Jahre (Median; IQR)	40,5 (32–49)	–
Weibliches Geschlecht	107	49,1
*Berufsgruppe*		
Pflegepersonal	108	50
Ärztliches Personal	98	45
Rettungsdienst	2	1
Sonstige	7	3
*Arbeitsbereich*		
Intensivstation	181	83
Intermediate-Care-Einheit	52	24
Notfallaufnahme/ZNA	29	13
Prähospitaler Rettungsdienst	16	7
OP	14	6
PACU/Aufwachraum	6	3
Sonstiges	15	7
*Fachrichtung*		
Intensivmedizin	83	38
Innere Medizin	67	31
Anästhesie	20	9
Interdisziplinär	16	7
Notfallversorgung	11	5
Operative Medizin	5	2
Pädiatrie/Neonatologie	1	1
Sonstiges	10	5
Keine Antwort	5	2
*Träger des Krankenhauses*		
Öffentlich	150	69
Freigemeinnützige	42	19
Private	19	9
Privatklinik	1	1
Keine Antwort	6	3

### Selbsteinschätzung zur Nachhaltigkeit

**Figure 2:**
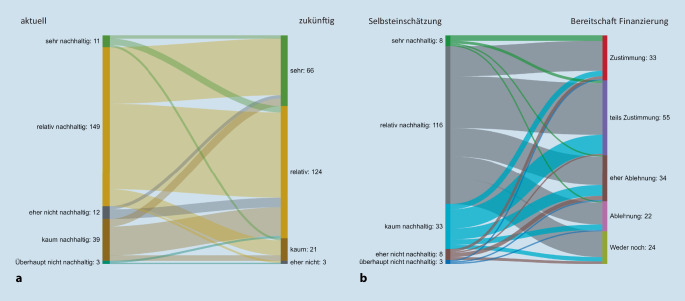

Unter den Teilnehmenden schätzen 149 (68 %) ihren aktuellen eigenen Lebensstil als „relativ nachhaltig“ ein. 124 (57 %) bzw. 66 (30 %) gaben an, den eigenen Lebensstil zukünftig „relativ“ bzw. „sehr nachhaltiger“ zu gestalten (Abb. 2). 88 Teilnehmende (50 %) gaben die Bereitschaft an, höhere Beiträge zur Gesundheits- und Krankenkasse zu zahlen, um eine verbesserte Nachhaltigkeit in der Gesundheitsversorgung zu erreichen (Abb. 2). 179 (82 %) der Teilnehmenden glauben, dass Nachhaltigkeit zu höheren Kosten führen wird, und geben diese höheren Kosten im Durschnitt mit 31 % an.

Der Glaube, dass man selbst Einfluss darauf hat, seinen Arbeitsbereich nachhaltiger zu machen, lag bei 47 % („stimme eher zu“: 80, [36,7 %] und „stimme voll zu“: 23, [10,5 %]), der Anteil der Unentschlossenen lag bei 35 (16 %) Teilnehmenden.

### Nachhaltigkeit an den Arbeitsstätten der Teilnehmenden

104 (47 %) Teilnehmende gaben an, dass ihre Arbeitsstätte Maßnahmen zur Nachhaltigkeit umgesetzt hat. 95 (43 %) Teilnehmende gaben an, dass keine Maßnahmen umgesetzt worden sind, diese sich aber in Planung befinden, oder noch keine Maßnahmen geplant oder umgesetzt wurden (47 [21 %] und 48 [22 %]).

Zu den konkreten Maßnahmen des Krankenhauses gaben die Teilnehmenden in erster Linie Maßnahmen aus dem Bereich der Mobilität (z. B. Jobticket, Ladesäulen für E‑Bikes etc.) an.

Als mögliche Motivation zur Umsetzung von Maßnahmen für Nachhaltigkeit in ihrem Betrieb gaben 91 (41,7 %) der Teilnehmenden eine Kostenreduktion an, 69 (31,7 %) sahen die Gründe in einer bestehenden Ressourcenknappheit, 57 (26 %) in gesellschaftlichem Druck und 32 (14,7 %) in politischem Druck. 8 (3,7 %) Teilnehmende glauben, dass die Motivation des Krankenhauses nur aufgrund der Initiative von Mitarbeitenden zustande gekommen ist.

### Organisationsstrukturen und Entscheidungsträger:innen

**Figure 3:**
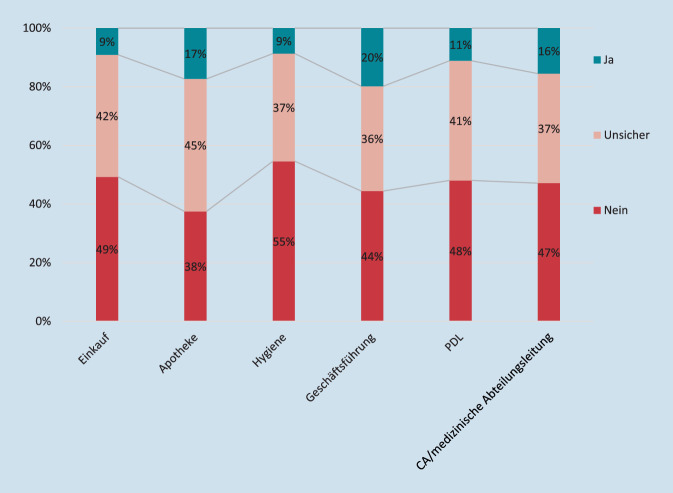

Bei der Frage, ob die Teilnehmenden glauben, dass die Organisationsstrukturen bzw. Entscheidungsträger:innen im Krankenhaus das Thema Nachhaltigkeit bei ihren Entscheidungen beachten, erhielt die Geschäftsführung mit 20 % den höchsten Wert; am schlechtesten wurden die Krankenhaushygiene und die Einkaufsabteilung mit 9 % bewertet (Abb. 3). Es wurden für die Befragung nur die Mitarbeiter befragt und nicht die entsprechenden Abteilungen.

### Allgemeine Nachhaltigkeit im eigenen Arbeitsbereich

**Figure 4:**
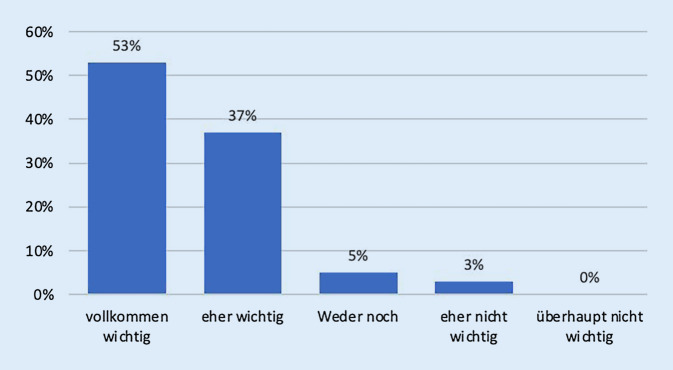

196 (90 %) Teilnehmende halten Nachhaltigkeit in ihrem Arbeitsbereich für „vollkommen wichtig“ bzw. „eher wichtig“ (Abb. 4).

44 (20 %) Teilnehmende gaben an, dass es in ihrem Arbeitsbereich Ansätze oder Modelle für Nachhaltigkeit gibt, wohingegen fast 80 % der Befragten keine Ansätze oder Modelle von Seiten der Krankenhausleitung sehen (143 [66 %]) bzw. nicht wissen, ob es diese Ansätze oder Modelle gibt (31 [14 %]).

Als bestehende Projekte im eigenen Arbeitsbereich werden folgende genannt (*n* = 41): Energiemanagement (11 [27 %]), Abfallmanagement (9 [22 %]), Schaffung von Nachhaltigkeitsstrukturen (8 [19 %]), Reduktion von Papiermüll sowie Management von volatilen Anästhetika (beides 6 [15 %]) und Reduktion von Einwegmaterial (5 [12 %]).

118 Teilnehmende gaben an, wo es in ihrem Bereich noch großes Potenzial für Nachhaltigkeit gibt: 63 (53 %): Abfallmanagement, 43 (36 %): Reduktion von Einwegmaterialien, 42 (36 %): Energiemanagement, jeweils 8 (7 %): Management von volatilen Anästhetika und besseren Mobilitätsmaßnahmen sowie 4 (3 %): Reduktion von Papiermüll.

197 (90 %) Teilnehmende gaben an, dass eine multiprofessionelle Zusammenarbeit für die Verbesserung der Nachhaltigkeit als „sehr wichtig“ einzuschätzen ist.

### Spezielle Nachhaltigkeit am eigenen Arbeitsplatz

#### Haltbarkeit

Bei Verbrauchsmaterialien (Verbandsmaterial, Spritzen etc.) würden 162 (74 %) der Teilnehmenden aktuell die Haltbarkeitsangaben der Hersteller weiter einhalten und die Materialien nicht zugunsten der Nachhaltigkeit über das angegebene Haltbarkeitsdatum hinaus verwenden wollen. Zukünftig würden dies nur noch 92 (42 %) der Teilnehmenden machen. Dieselbe Einschätzung zu Einmalmaterialien (Scheren, Klemmen etc.) teilten aktuell 130 (60 %) Teilnehmende und in Zukunft 69 (32 %) Teilnehmende, bei Trägerlösungen aktuell 175 (80 %) und zukünftig 121 (56 %), bei selbsthergestellten Infusionslösungen aktuell 172 (79 %) und zukünftig 134 (61 %).

#### Bettplatzaufbereitung

82 (38 %) Teilnehmende gaben an, dass zur Bettplatzaufbereitung nach Entlassung einer/s Isolationspatient:in das komplette Material aus dem Zimmer bzw. vom Bettplatz entsorgt wird. 116 (53 %) gaben an, dass je nach Isolationsstufe das Material entsprechend entsorgt wird.

### Mögliche Nachteile durch Nachhaltigkeit

188 (86 %) Teilnehmende sahen weder für sich noch für die Patient:innen eine Gefahr durch nachhaltigeres Arbeiten. In den Fällen, wo eine Gefahr gesehen wurde, war ursächlich bei jeweils 14 bzw. 13 Teilnehmenden die Sorge vor hygienischen Mängeln und Infektionsgefahr für die Patient:innen. 10 Teilnehmende sahen eine Infektionsgefahr für sich selbst oder man sah den persönlichen Schutz gefährdet. 8 Teilnehmende gaben an, dass möglicherweise eine Gefahr für die Patient:innen durch ein höheres Arbeitsaufkommen (komplexere Arbeitsabläufe etc.) entstehen könnte.

## Diskussion

Der Wunsch und der Wille der Mitarbeitenden der Intensivstationen und Notaufnahmen, das Thema Nachhaltigkeit anzugehen und Maßnahmen umzusetzen, ist sehr groß, wie die Ergebnisse dieser Umfrage mit  90 % Zustimmung zeigen. National oder international gibt es kaum vergleichbare Studien zu diesem Thema.

In unserer Umfrage gaben nur 20 % der Teilnehmenden an, dass es konkrete Ansätze für Nachhaltigkeit in ihrem Einsatzbereich gibt. Die Einschätzung, ob bestimmte Organisationsstrukturen und Entscheidungsträger:innen im Krankenhaus das Thema Nachhaltigkeit in ihren Entscheidungen beachten, wurde ebenfalls lediglich mit 9–20 % angegeben. Trotz der Anzeichen also, dass die Motivation vorhanden ist, zeigen die Umfrageergebnisse auch, dass dieses „heiße Thema“ bislang kaum von Seiten der Krankenhausleitungen angegangen wurde oder dass aufgrund mangelhafter oder fehlender Kommunikation bereits etablierte Ansätze durch die Mitarbeitenden nicht wahrgenommen werden.

Dies steht teilweise auch im Einklang mit der aus Deutschland kommenden Arbeit von Seifert et al. Dort wurden verschiedene Interessengruppen (Vorstand, Mitarbeitende etc.) von Krankenhäusern gefragt, für wie wichtig sie die Implementierung von Maßnahmen für mehr Nachhaltigkeit einschätzen. Die Ergebnisse zeigen, dass die Akteure durch unterschiedlichen externen Druck sehr unterschiedliche Interessen haben und zusätzlich teilweise nur wenige Kenntnisse über Nachhaltigkeit haben. In der Interpretation der Studienergebnisse wurde zusammengefasst, dass gerade diese unterschiedlichen Intentionen der Entscheidungsträger:innen die nachhaltige Entwicklung im Gesundheitswesen behindern und dass es entscheidend ist, dass alle Akteure über mögliche Maßnahmen informiert werden und die Interessen aller Akteure eines Krankenhauses in Bezug auf nachhaltiges Arbeiten evaluiert werden [13].

Eine aktuelle Arbeit von Quitmann et al. aus Deutschland hat in einer kleinen Studie mittels semistrukturierten qualitativen Experteninterviews herausgearbeitet, dass die Abschwächung des Klimawandels im Allgemeinen zwar als wichtig angesehen wurde, aber sowohl Patient:innen als auch leitende Verantwortliche sich weniger verantwortlich für die Eindämmung des Klimawandels gesehen haben. Die bestmögliche medizinische Versorgung wurde in dieser Untersuchung als oberste Priorität angegeben. Die Befragten waren oft besorgt darüber, dass die Gesundheit der Patient:innen durch Maßnahmen zur Eindämmung des Klimawandels beeinträchtigt werden könnte [11]. Unsere diesbezügliche Frage konnte dies nicht bestätigen. 86 % der Befragten sahen weder für sich noch für die Patient:innen eine Gefahr durch mehr nachhaltigeres Arbeiten im Intensiv- und Notfallsetting. Unsere Ergebnisse und die erwähnten Vorstudien deuten auf die Notwendigkeit einer Optimierung der Organisationskultur in Krankenhäusern hin. Die oftmals als Unternehmen geführten Kliniken müssen beim Thema Nachhaltigkeit ihrer gesellschaftlichen Verpflichtung nachkommen und die Mitarbeitenden in ihren Bedürfnissen unterstützen. Hierfür ist nicht nur eine ausreichende Verbindlichkeit von Seiten der Krankenhausleitungen nötig, sondern Nachhaltigkeit muss integraler Bestandteil der Kultur des Krankenhauses sein.

Größtes Potenzial für eine bessere Nachhaltigkeit im eigenen Arbeitsbereich wurde unter anderem im Abfallmanagement gesehen. Diese Ergebnisse überlappen mit denen in einer Untersuchung von Hunfeld et al. In dieser Studie wurde eine Materialflussanalyse bei 2839 Intensivpatien:innen durchgeführt. Die sehr gute und akribisch durchgeführte Arbeit zeigt erstmals Daten u. a. zur Menge an produziertem Abfall (247.000 kg für das Untersuchungsjahr 2019, davon 50.000 kg Isolationsmüll). Die meisten Umweltbelastungen wurden auf der untersuchten Intensivstation durch die Verwendung von Alltagsmaterialien verursacht und nicht durch den Materialverbrauch für bestimmte Therapien (Beatmung, Nierenersatztherapie oder ECMO). Es konnten 5 Einmalartikel ermittelt werden, die für eine Kreislaufwirtschaft (im Sinne einer Wiederverwertung) besonders interessant sind: unsterile Handschuhe, Isolierkittel, Bettbezüge, chirurgische Masken und Spritzen [5].

In unserer Umfrage gaben 27 % der Teilnehmenden an, dass in ihrem Bereich bereits Maßnahmen zur Verbesserung des Energiemanagements umgesetzt wurden, und 36 % sahen in ihrem Bereich großes Potenzial in diesem Bereich.

In einer schon 2014 publizierten Arbeit von Pollard et al. wurde der mittlere Energieverbrauch einer/s Intensivpatient:in pro Tag ermittelt. Hauptsächlich für die Betreuung der/Intensivpatien:innen und die Überwachung ihres Zustands wurden damals 15 kWh/Tag Strom benötigt (ohne Verbrauchsartikel); dies entspricht dem Verbrauch eines durchschnittlichen 4‑Personen-Haushalts [10]. 2018 untersuchte die Arbeitsgruppe von McGain in den USA den Energieverbrauch von 10 septischen Patient:innen. Der durchschnittliche Energieverbrauch ergab einen Wert von 272 kWh/Tag (inklusive Verbrauchsmaterialien, Medikamente etc.) und Patient:in [7].

Insgesamt bleibt jedoch einschränkend festzuhalten, dass die Ökobilanz einer Intensivstation sich aus verschiedensten Produkten und Prozessen (Verbrauchsmaterialien, Medikation, Energie, Personal usw.) zusammensetzt. Die Analyse der Ökobilanz hängt daher auch von regionalen und lokalen Faktoren (z. B. Anteil an fossilen Energieträgern) ab und ist schwer zu verallgemeinern [3].

Zusammenfassend kann man folgende Kernaussagen aus der Umfrage ableiten:

1. Es besteht eine hohe Motivation der Mitarbeitenden, sich mit dem Thema Nachhaltigkeit auseinanderzusetzen und Maßnahmen umzusetzen.
2. Das Potenzial, ein ressourcenschonendes und umweltfreundliches Krankenhaus zu etablieren, ist längst nicht ausgeschöpft.
3.  Es muss Priorität werden, dass Entscheidungsträger:innen Nachhaltigkeit propagieren, Prozesse transparent gestalten und die Motivation der Mitarbeitenden zum Thema Nachhaltigkeit unterstützen.

Maßnahmen für eine nachhaltige und ressourcenschonende Intensiv- und Notfallversorgung können durch folgende Aspekte erreicht werden:

1. Energieeffizienz: Energieeffiziente Geräte und Verfahren können den Energieverbrauch reduzieren.
2. Vermeidung von unnötigen Eingriffen: Die Vermeidung unnötiger Diagnostik und Therapie kann dazu beitragen, den Ressourcenbedarf auf der Intensivstation zu minimieren.
3. Verwendung von recyclingfähigen Materialien: Dies kann dazu beitragen, den Abfallaufwand zu reduzieren und die Umweltbelastung zu verringern. Es sollte von Beginn an darauf geachtet werden, die Artikel in eine Kreislaufwirtschaft einzubinden.
4. Implementierung einer Stabsstelle Nachhaltigkeit im Krankenhaus, die als Knotenpunkt der Kommunikation mit den einzelnen Entscheidungsträger:innen im Krankenhaus fungiert.
5. Bildung und Schulung zu Nachhaltigkeit: Dies kann dazu beitragen, dass Ärzt:innen und Pflegefachpersonen sich für nachhaltige Praktiken engagieren und ihre Verantwortung für die Umwelt und den Ressourcenschutz erkennen und in ihren Alltag implementieren

Ein erster Schritt für die Einleitung der Maßnahmen kann die Bildung von lokalen „grünen Teams“ sein, die in der jeweiligen Abteilungen federführend für die Planung, Umsetzung und auch Beurteilung von Maßnahmen sind [15]. Zusätzlich können bereits bestehende Manuals, wie das „A beginners guide to sustainability in the ICU“ der Australian and New Zealand Intensive Care Society (ANZICS), mit konkreten Empfehlungen bei der Einleitung von Maßnahmen hilfreich sein [1].

Die Studie hat mehrere Limitationen: Der Rücklauf der Umfrage ist eher geringer ausgefallen als erwartet. Es können keine prozentualen Angaben zur Rücklaufquote gemacht werden, da aufgrund des offenen Einladungsmodus keine quantitative Zählung der Empfänger durchgeführt werden konnte. Aus einigen Bundesländern gab es kaum bzw. keine Teilnehmenden. Es ist denkbar, dass hier auch die gesamtpolitische Lage im jeweiligen Bundesland das Denken und die Beteiligung zum Thema Nachhaltigkeit beeinflusst haben.

Zum anderen ist davon auszugehen, dass der Großteil der Befragten an einem Krankenhaus der Maximalversorgung bzw. einer Uniklinik arbeitet (44 % an einem Krankenhaus mit  1000 Betten). Es könnte sein, dass Aktivitäten zur Nachhaltigkeit in Krankenhäusern mit geringerer Bettenkapazität anders aussehen und damit hier nicht repräsentativ darzustellen sind. Zudem besteht ein unspezifischer Selektionsbias dadurch, dass Personen, die sich ohnehin mit dem Thema beschäftigen, sich auch von einer Umfrage zur Thematik Nachhaltigkeit angesprochen fühlen. In dieser ersten bundesweiten Umfrage sind auch keine Fragen bezüglich einer Vermeidung von unnötigen Eingriffen bzw. intensivmedizinischen Überwachungsindikationen eingegangen. Auch die Diskussion über die Folgen einer Übertherapie bezüglich Nachhaltigkeit wurde nicht aufgenommen. Diese ethisch-moralischen Aspekte von Intensiv- und Notfallversorgung sollen in einer weiteren Umfrage bearbeitet werden.

Die Nachhaltigkeit in der Intensiv- und Notfallmedizin muss ein laufender Prozess, der sich ständig weiterentwickelt, werden, denn die Anforderungen an das Gesundheitssystem werden immer herausfordernder.

Die Ergebnisse dieser Umfrage können helfen, ein besseres Verständnis für die Einstellungen und Meinungen in Bezug auf Nachhaltigkeit zu erlangen und darauf aufbauend Maßnahmen zur Förderung einer nachhaltigeren Intensiv- und Notfallversorgung zu entwickeln.

## Fazit für die Praxis

- Der Gesundheitssektor ist für 5–7 % der CO_2_-Emissionen verantwortlich. Die Intensivstationen und Notaufnahmen tragen zu diesen CO_2_-Emissionen einen erheblichen Anteil bei.
- Die Umfrageergebnisse zeigen, dass eine hohe Motivation der teilnehmenden Mitarbeitenden besteht, die Zukunft ressourcenschonend und umweltbewusst zu gestalten. Es gilt insbesondere, die Entscheidungsträger:innen in den Krankenhäusern in die Pflicht zu nehmen und auch Politik und Gesundheits- und Krankenkassen um Unterstützung zu bitten. Es wird weder eine Gefahr für das Personal noch für Patient:innen durch ein nachhaltigeres Handeln gesehen.
- Das Thema Nachhaltigkeit sollte in die Denkkultur eines jeden Krankenhauses implementiert werden und es ist dringend an der Zeit, konkrete Maßnahmenpläne zu erarbeiten.
